# Measuring Job Crafting Across Cultures: Lessons Learned From Comparing a German and an Australian Sample

**DOI:** 10.3389/fpsyg.2019.00991

**Published:** 2019-05-07

**Authors:** Vivian Schachler, Sandra D. Epple, Elisa Clauss, Annekatrin Hoppe, Gavin R. Slemp, Matthias Ziegler

**Affiliations:** ^1^Department of Psychology, Humboldt-Universität zu Berlin, Berlin, Germany; ^2^Department of Psychology and Ergonomics, Technische Universität Berlin, Berlin, Germany; ^3^Confederation of German Employers’ Associations (BDA), Berlin, Germany; ^4^Melbourne Graduate School of Education, The University of Melbourne, Parkville, VIC, Australia

**Keywords:** job crafting, psychometric properties, measurement invariance, reliability, JCQ

## Abstract

Job crafting refers to the act of employees actively altering work aspects to better suit their values and interests. Slemp and Vella-Brodrick (2013) proposed a Job Crafting Questionnaire (JCQ) in English consisting of three facets: task crafting, cognitive crafting, and relational crafting. This is in line with the original conceptualization of job crafting by Wrzesniewski and Dutton (2001). However, there has not yet been an evaluated German translation of this measure. Therefore, this paper aims at evaluating the psychometric properties of scores from a German translation of the JCQ, using the original Australian dataset and a German sample of 482 employees. Our findings showed first evidence for the reliability and validity of the scores. We also extend prior research and include creative self-efficacy in the nomological network of job crafting. Importantly, strong factorial measurement invariance was demonstrated, allowing for comparisons between the job crafting scores of German- and English-speaking samples. Based on this example, we highlight the importance of enriching measurement invariance tests by including other key constructs. Our results suggest that the German JCQ is an acceptable tool for measuring job crafting, as originally conceptualized by Wrzesniewski and Dutton (2001).

## Introduction

For a long time, employees were seen as passive recipients of their work environment (Loher et al., 1985) despite them having the opportunity to actively influence their working experience (Wrzesniewski and Dutton, 2001). One way of doing so is to engage in job crafting – a type of proactive work behavior (Grant and Ashford, 2008) that involves employees initiating changes employees to redesign their work tasks (i.e., task crafting), their thoughts about their work (i.e., cognitive crafting), and their relationships at work (i.e., relational crafting) in order to fulfill their personal interests and needs (Wrzesniewski and Dutton, 2001). Thus, job crafting is a potentially important aspect in organizational assessments or transformation processes. So far, most measures that capture job crafting are in English, which severely limits cross-cultural research. The current project aimed at closing this gap by developing and evaluating a German translation of the original Job Crafting Questionnaire (JCQ, Slemp and Vella-Brodrick, 2013). Moreover, we will highlight the importance of including parts of the nomological network in the measurement invariance testing procedure. Specifically, we expect to derive further information regarding measurement invariance from this by testing whether the correlation between job crafting and job satisfaction is equal.

### The Construct of Job Crafting

Job crafting refers to changes actively initiated by employees to alter physical, cognitive, or social aspects of their work to better suit their values and interests (Slemp and Vella-Brodrick, 2013). Wrzesniewski and Dutton (2001) introduced a theoretical job crafting model which was later empirically tested (e.g., Berg et al., 2008; Lyons, 2008). Wrzesniewski and Dutton (2001) proposed three different forms of job crafting – which involves physically or cognitively altering task boundaries or influencing relational boundaries – that employees face on their job: task, cognitive, and relational crafting. *Task crafting* involves changing the type or the number of activities at work. *Cognitive crafting* refers to changes in the way individuals perceive their job with respect to its meaningfulness. Finally, *relational crafting* concerns the choice of social contacts at work, the kinds of relationships sought, and the amount of time and energy invested in these relationships (Wrzesniewski and Dutton, 2001; Slemp and Vella-Brodrick, 2013).

### The Nomological Network of Job Crafting

Wrzesniewski and Dutton (2001) described several important antecedents (i.e., motivation to job craft) and consequences (i.e., changes in job design and meaning) of job crafting as well as moderators (i.e., perceived opportunities to craft one’s job) of these relations. This theoretical model sparked considerable empirical research aimed at testing the proposed relations (e.g., Berg et al., 2008; Lyons, 2008; Slemp and Vella-Brodrick, 2013, 2014; Niessen et al., 2016) and at uncovering other constructs related to job crafting.

Elaborations about the job crafting model and other associated constructs can be translated into a nomological network of job crafting (Cronbach and Meehl, 1955). We will use the relations specified in this nomological network to formulate concrete hypotheses (Ziegler, 2014). These hypotheses test whether job crafting scores from the German version of the job crafting measure have relations to scores that operationalize other constructs and are in line with the theoretical assumptions about job crafting and its associated constructs. To achieve this, we tested parts of the nomological network using a selection of similar, antecedent, and consequent constructs, and subsequently integrated this into the measurement invariance testing procedure below.

#### Similar Constructs

A construct similar to job crafting is personal initiative, which is defined as “taking an active and self-starting approach to work and going beyond what is formally required in a given job” (Frese et al., 1997, p. 140). People act proactively and take initiative to improve their current work context (Crant, 2000). Similarly, job crafting is understood to be one type of proactive behavior and implies that employees take initiatives to alter aspects of their job for the better (Grant and Ashford, 2008; Slemp and Vella-Brodrick, 2013). As this is a similar construct, we expected a moderate correlation. Importantly, however, it is not the same construct, thus the expected correlation was more in the sense of a discriminant correlation.

#### Antecedents

According to Wrzesniewski and Dutton (2001), autonomy-supportive work contexts moderate the relationship between job crafting motivation and actual job crafting behavior. Autonomy makes it easier for employees to craft their job. Niessen et al. (2016) showed positive correlations between job crafting and job autonomy scores.

Berg et al. (2010) found that employees directed job crafting behaviors toward aspects of their work where they felt effective. Niessen et al. (2016) showed small correlations between self-efficacy and job crafting. Self-efficacy allows employees to act in a more self-initiated and proactive manner due to their belief in personal success (Parker et al., 2010). Wrzesniewski and Dutton (2001, p. 180) described job crafting as a “creative and improvised process that captures how individuals locally adapt their jobs.” Therefore, they see job crafters as shaping their job in a creative way. In line with this, it seems plausible that creativity plays a role in the nomological network of job crafting. For this reason and taking into account Bandura’s theory of self-efficacy (Bandura and Walters, 1977), we focus on a domain-specific conceptualization of self-efficacy, namely creative self-efficacy (Tierney and Farmer, 2002). Creative self-efficacy reflects “employees’ beliefs in their ability to be creative in their work” (Tierney and Farmer, 2002, p. 1141). Again, we expected moderate relations.

#### Consequences

Vigorous employees experience high levels of energy and willingness to invest effort in their work (Schaufeli and Bakker, 2004). In line with the self-determination theory (Deci and Ryan, 2008), vigor can be described as a consequence of job crafting (Slemp, 2017). Because employees craft their job to satisfy their personal needs, they should feel more vigorous afterwards. Studies have shown that employees’ vigor is enhanced when their needs are satisfied (e.g., Van den Broeck et al., 2008).

Finally, literature has also linked job crafting to employees’ job satisfaction. Job satisfaction is described as “a global feeling about the job or as a related constellation of attitudes about various aspects or facets of the job” (Spector, 1997, p. 2). By engaging in job crafting, employees ultimately attain a more satisfying working situation – which positively links job crafting to job satisfaction (e.g., Ghitulescu, 2007; Berg et al., 2008; Slemp and Vella-Brodrick, 2013). Again, we expected moderate relations.

We expected all relations of job crafting and its similar, antecedent, and consequent construct scores to be positive.

### Measurement of Job Crafting and Measurement Invariance

Oldham and Fried (2016) explicitly demanded more research that compared the frequency of job crafting in different cultures. For this purpose, it is necessary to have a psychometrically tested scale of job crafting in different languages to be used in multicultural work settings (Ziegler and Bensch, 2013). Several English-language instruments measuring job crafting have been proposed in the literature (e.g., Ghitulescu, 2007; Leana et al., 2009; Tims et al., 2012; Slemp and Vella-Brodrick, 2013; Nielsen et al., 2017). Recently, two German measures of job crafting have been proposed (Lichtenthaler and Fischbach, 2016; Niessen et al., 2016). However, these scales either focus on a theoretical model that differs from the original three-facet conceptualization of job crafting by Wrzesniewski and Dutton (2001) (e.g., Tims et al., 2012; Lichtenthaler and Fischbach, 2016) or have not been tested for measurement invariance (e.g., Niessen et al., 2016). Therefore, comparisons between cultures using these measures could be biased (Chen et al., 2005; Chen, 2007).

So far only two studies have tested job crafting measures and found them to be invariant across languages and cultures (Gordon et al., 2015; Nielsen et al., 2017). Gordon et al. (2015) tested the job crafting measure of Petrou et al. (2012) and found it to be invariant across a Dutch and American sample. Multigroup analyses supported the assumption of weak factorial invariance. Nielsen et al. (2017) conducted a large study to test the psychometric properties and measurement invariance of the Job Crafting Questionnaire (JCRQ) based on Tims et al. (2012) in four different cultural samples (i.e., Spain, China, Taiwan, and the United Kingdom). The JCRQ showed weak factorial invariance, with the exception of the Chinese version which differed from the United Kingdom and Spanish versions of the JCRQ. At the moment there is no equivalent study for a job crafting measure based on the theory of Wrzesniewski and Dutton (2001) in the German language. Therefore, we explored whether the Job Crafting Questionnaire (JCQ) by Slemp and Vella-Brodrick (2013) measures the same construct, i.e., job crafting, across nationalities, i.e., in Germany and Australia, and allows for cross-cultural comparisons.

Importantly, the cited invariance tests only focused on the factorial structure of the measure. While this is currently the standard, we want to highlight here the advantages of also including the relation of job crafting with another measure in the procedure of invariance testing. Testing configural, weak, and strong factorial invariance ensures that latent correlations and means can be compared (Meredith, 1993). Clearly, this is an important prerequisite for cross-cultural research or other research that compares groups (Chen, 2008). However, whereas the formalities of testing measurement invariance have been laid out repeatedly (Sass, 2011), ensuing group comparisons are often exploratory in nature. We argue here that comparisons of relations with other measures should be part of the actual invariance testing. In this way, based on prior research and theory, mean or correlational differences or non-differences can be delineated. A confirmation of these assumptions would strongly support the assumed measurement invariance. In this paper we will exemplify this strategy.

#### Relation With Job Satisfaction

In addition to invariance tests and in line with the recommendations of the International Test Commission [ITC] (2017) guidelines, it seems plausible to compare key correlations derived from the nomological network of job crafting across cultures. This procedure helps us to assess construct equivalence across both versions of the JCQ. The relation between job satisfaction and job crafting is of importance. For job crafting to be feasible in the same way across countries, it seems important that the cultural values underlying work place behavior are assumed to be comparable. Prior research has shown that Australia (Anglo culture) and Germany (Germanic culture) are rather comparable to each other in terms of their cultural values (Ronen and Shenkar, 1985). Different cultural dimensions (Hofstede, 1980) have been shown to be related to job satisfaction (Kirkman and Shapiro, 2001). Especially masculinity (a doing orientation) is the cultural value related with job satisfaction. It has been shown that Australia and Germany do not differ much in terms of masculinity (Hofstede et al., 2010). Thus, we expect job crafting to address similar aspects in both countries and thereby show the same relation with job satisfaction. Testing this assumption in addition to classical invariance testing would show that the scores from the translated measure have comparable measurement properties in different countries. In particular, this strategy would show that not only the factorial structure is comparable but also that key theoretical assumptions of the model can be found across cultures. Importantly, as outlined below, there should not be cultural differences that alter the assumed key relation in one of the cultures under investigation. Thus, this addition to the classical procedure should further help to build trust in the translated measure.

We focused on job satisfaction because it is a key correlation derived from the nomological network of job crafting, and it is a construct that was operationalized in both data sets.

### Aims of the Current Study

The aims of this study were twofold. First, we aimed to provide a psychometrically sound German measure of job crafting that conforms to the original job crafting model conceptualized by Wrzesniewski and Dutton (2001). Second, we aimed to propose an extension for classical invariance testing and delineate the lessons learned. To achieve these aims, we translated the job crafting questionnaire (Slemp and Vella-Brodrick, 2013) and evaluated psychometric properties in three steps: (a) we evaluated specific correlations between job crafting scores and scores for related constructs in the nomological network; (b) we tested measurement invariance with the original scale, and (c) we tested whether the relation between job crafting and job satisfaction, a key criterion within the nomological network, was also invariant across the two cultures investigated in our study.

## Materials and Methods

### Participants

The analyses for this study were based on two sub-samples, namely a German sample and the Australian sample used by Slemp and Vella-Brodrick (2013). The German sample was recruited for a larger research project, which examined employees working in individualized working conditions and consisted of *n* = 482 participants. The data were collected from January to April of 2015. Participants were recruited through emails (e.g., participants from other studies who allowed us to inform them about new studies), flyers (e.g., handed out in different organizations, to participants at workshops, or to persons in our private networks), newspaper advertisements, and internet platforms (e.g., different Facebook groups, project websites for occupational health). Upon contacting us, participants received an email containing information about the study (aim, benefits, general conditions, confidentiality of data, voluntary nature of participation) as well as a link to the website of the study and the questionnaire. To obtain answers to our research questions, we aimed to recruit adult employees who were (a) flexible in working hours, (b) able to organize their work individually, and/or (c) who had managerial functions. These inclusion criteria were only mentioned in the study information the employees received. We did not have any filter variables regarding these criteria. To be included, it was only necessary that the participants were in paid employment. We did not exclude any participant who completed the questionnaire. All participants had at least one job. All types of employment and job positions were eligible. Completion of the questionnaire comprised their informed consent for their participation. The participants were not compensated monetarily. The consent procedure and study protocol received ethical approval from the ethics committee of the Institute of Psychology of Humboldt-Universität zu Berlin, Germany. The participants’ age ranged from 19 to 72 (*M* = 37.87, *SD* = 10.68) with 36% of participants male, 59% female, and 5% did not provide any information regarding gender. Furthermore, 7% of participants finished their education with vocational training; 12% had earned a university-entrance diploma (Abitur, A Levels, or equivalent); 7% of participants had attended an undergraduate program (Bachelor or equivalent), and 44% had earned a Master’s degree or equivalent, and 17% a Ph.D. Moreover, 83% of participants indicated that they worked full time; 17% worked part -time. On average, the participants worked 42.06 h per week (*SD* = 12.21). In the sample, 35% of participants worked in education and research, 32% in journalism and media, 7% in the service industry, and 5% in health care.

A second measurement wave was conducted 4 weeks after the first assessment. Of the 482 participants who completed the first questionnaire, *n* = 123 also participated in the second measurement wave (*M*_age_ = 36.29, *SD*_age_ = 10.19, 62% female). In this study, we used the second measurement wave for test–retest reliability estimation only. There were no significant differences between the samples in the first and second measurement waves regarding gender and all psychological constructs except for task crafting, relational crafting, personal initiative, and age (see Appendix A). Moreover, a logistic regression analysis was conducted to predict drop-out at the second measurement wave using age, gender, and all psychological constructs as predictors. None of the regression coefficients was significant and Nagelkerkes’s *R*^2^ was 0.026, indicating that none of the mentioned constructs predicted drop-out at the second measurement wave.

The Australian sample contained data of *n* = 334 employees. Of these 334 employees, only 253 reported their demographics. In this study, we analyzed existing data from a battery of questionnaires that was administered online to employees in Australia in 2011. This sample was recruited through social networking sites (e.g., Facebook), online discussion forums (e.g., about positive psychology), and through staff email and newsletters of organizations. To be included, all participants had to be at least 18 years of age and had to be in paid employment. In accordance with the German sample, there were also no additional exclusion criteria. Our reasoning was that anyone in any type of job would have at least some capacity to craft their job, regardless of their level, occupation, or experience, and so we included all participants who completed the questionnaire. Participants were directed to an explanatory statement which contained a link to the online battery of questionnaires. Participation in this study was voluntary. Implied consent was used– i.e., after reading the explanatory statement, if the participants agreed to what was involved they then went on to complete the survey. All procedures were approved by the Monash University Human Research Ethics Committee (MUHREC), Australia, in December 2010. The participants’ age ranged from 23 to 71 (*M* = 41.95, *SD* = 11.36) with 32% of participants male and 68% female. In the Australian sample, educational background was assessed via the years of formal education. The participants had received between 8 and 33 years of formal education (*M* = 17.59, *SD* = 3.57). Moreover, 74% worked full time, and 26% worked part time. On average, the participants worked 37.95 h per week (*SD* = 12.13). In the sample, 68% of participants worked in education, 6% in financial services, and 6% in health care. A table comparing the sociodemographic variables of the German and Australian samples can be found in Appendix B. There was no significant difference between the German and Australian samples concerning gender composition. However, the German and the Australian samples differed significantly regarding the variables age, employment type, and working hours per week (see Appendix B).

### Measures

The descriptive statistics and reliability estimates of all measures used can be found in Table 1.

**Table 1:** Descriptive statistics and correlations between job crafting and associated measures.

					Reliability													
	*M^1^*	*SD^1^*	*M^2^*	*SD^2^*	α^1^	α^2^	ω^1^	ω^2^	test--retest (*r*)^1^	Job crafting	Task crafting	Cognitive crafting	Relational crafting	Personal initiative	Autonomy	Creative self-efficacy	Vigor	Job satisfaction
Job crafting	3.24	0.47	3.74	0.94			0.66	0.79	0.71^∗∗^		0.83^∗∗^ (0.79–0.86)	0.83^∗∗^ (0.79–0.86)	0.78^∗∗^ (0.74–0.82)					0.43^∗∗^ (0.34–0.51)
Task crafting	3.30	0.56	3.82	1.14	.70	0.86	0.78	0.89	0.68^∗∗^	0.69^∗∗^ (0.64–0.73)		0.54^∗∗^ (0.46–0.61)	0.46^∗∗^ (0.37–0.54)					0.38^∗∗^ (0.29–0.47)
Cognitive crafting	3.28	0.68	3.70	1.21	0.76	0.89	0.75	0.91	0.63^∗∗^	0.75^∗∗^ (0.71–0.78)	0.27^∗∗^ (0.19–0.35)		0.45^∗∗^ (0.36–0.53)					0.45^∗∗^ (0.36–0.53)
Relational crafting	3.11	0.72	3.68	1.13	0.73	0.84	0.74	0.85	0.65^∗∗^	0.71^∗∗^ (0.67–0.75)	0.29^∗∗^ (0.21–0.37)	0.29^∗∗^ (0.20–0.37)						0.21^∗∗^ (0.11–0.31)
Personal initiative	3.87	0.55			0.79					0.42^∗∗^ (0.35–0.49)	0.33^∗∗^ (0.25–0.41)	0.27^∗∗^ (0.19–0.35)	0.34^∗∗^ (0.26–0.42)					
Autonomy	3.78	0.96			0.86					0.19^∗∗^ (0.10–0.27)	0.25^∗∗^ (0.17–0.34)	0.06 (−0.03–0.14)	0.10^∗^ (0.01–0.18)	0.11^∗^ (0.02–0.20)				
Creative self-efficacy	3.99	0.66			0.73					0.32^∗∗^ (0.23–0.39)	0.31^∗∗^ (0.23–0.39)	0.22^∗∗^ (0.13–0.30)	0.18^∗∗^ (0.09–0.26)	0.55^∗∗^ (0.49–0.61)	0.16^∗∗^ (0.07–0.25)			
Vigor	4.29	1.61			0.86					0.39^∗∗^ (0.31–0.46)	0.31^∗∗^ (0.23–0.39)	0.29^∗∗^ (0.21–0.37)	0.26^∗∗^ (0.17–0.34)	0.35^∗∗^ (0.27–0.43)	0.15^∗∗^ (0.06–0.24)	0.30^∗∗^ (0.22–0.38)		
Job satisfaction	5.34	1.37							0.73^∗∗^	0.28^∗∗^ (0.19–0.36)	0.24^∗∗^ (0.15–0.32)	0.18^∗∗^ (0.09–0.26)	0.20^∗∗^ (0.11–0.28)	0.17^∗∗^ (0.08–0.26)	0.21^∗∗^ (0.12–0.29)	0.19^∗∗^ (0.10–0.27)	0.63^∗∗^ (0.57–0.68)	

*α, Cronbach’s alpha; ω, McDonald’s omega; r, Pearson correlation coefficient. The 95% confidence intervals are shown in the brackets below the correlation estimates. Correlations within the German dataset are displayed below the diagonal; correlations within the Australian dataset are shown above the diagonal.^1^German dataset (5-point Likert-type scale), ^2^Australian dataset (6-point Likert-type scale). ^∗^p < 0.05, ^∗∗^p < 0.01.*

#### Job Crafting Questionnaire

The JCQ by Slemp and Vella-Brodrick (2013) consists of 15 items. Each of the three job crafting facets (task, cognitive, and relational crafting) was measured using five items. The original rating format was a 6-point Likert-type scale ranging from 1 (hardly ever) to 6 (very often). In accordance with guidelines proposed by Schmitt and Eid (2007) and the guidelines of the (International Test Commission [ITC], 2017), the German translation of the JCQ was obtained using the translation–retranslation method conducted by native German speakers and English-speaking professionals, respectively. The German version contained a 5-point Likert-type scale (1 = never to 5 = very often). This different rating format was used because the dataset was collected for a different research purpose and the researchers wanted to simplify the filling-in process of the questionnaire by providing consistent rating scales across measures. All original job crafting items and all corresponding German items can be found in Appendix C.

#### Personal Initiative

To measure personal initiative, we used a questionnaire proposed and evaluated by Frese et al. (1997). The scale measures self-reported initiative with seven items (e.g., “I actively attack problems.”) using a 5-point Likert-type scale ranging from 1 (totally disagree) to 5 (totally agree). Frese et al. (1997) confirmed the good psychometric properties (e.g., reliability and evidence of validity) of the original German version of the scale.

#### Autonomy

To assess autonomy, a German version of the Work Design Questionnaire (WDQ, Stegmann et al., 2010) proposed by Morgeson and Humphrey (2006) was used. We only used the facet of the task characteristics that captures work-scheduling autonomy. This subscale consists of three items (e.g., “The job allows me to plan how I do my work.”) rated on a 5-point Likert-type scale ranging from 1 (totally disagree) to 5 (totally agree). The internal consistency of the facet was reported to be adequate. Evidence for the validity of the MDQ was supported by evaluations of relations with other variables (Morgeson and Humphrey, 2006). The German translation of the WDQ was evaluated and found to have good psychometric properties by Stegmann et al. (2010).

#### Creative Self-Efficacy

The German translation of the creative self-efficacy scale proposed by Tierney and Farmer (2002, 2011) was used. It consists of three items (e.g., “I feel that I am good at generating novel ideas.”) which are rated on a 5-point Likert-type scale ranging from 1 (strongly disagree) to 5 (strongly agree). Tierney and Farmer (2002) provided support for its good internal consistency and presented various evidence supporting the validity of the measure. To our knowledge, the scale has not been evaluated in the German language yet.

#### Vigor

To assess vigor, we used the German translation of the short version of the Utrecht Work Engagement Scale (UWES-9, Schaufeli et al., 2006). We administered the vigor scale of the questionnaire which consists of three items (e.g., “At my work, I feel bursting with energy.”) rated on a 7-point Likert-type scale ranging from 1 (never) to 7 (always). Schaufeli et al. (2006) found evidence for its validity with regard to the internal structure of the UWES-9 and demonstrated adequate reliability in 10 different countries (e.g., Germany).

#### Job Satisfaction

We assessed global job satisfaction using a single-item measure (“How satisfied are you with your work in general?”) with a 7-point Likert-type scale ranging from 1 (very dissatisfied) to 7 (very satisfied). Single-item job satisfaction measures (e.g., the Kunin Scale) have often been used in the past (Wanous and Reichers, 1996; Wanous et al., 1997). They have been shown to be acceptable for investigating overall job satisfaction, demonstrating reasonable reliability as well as evidence for validity based on the internal structure and relations to other variables.

In the Australian sample job satisfaction was assessed using the Michigan Organizational Assessment Questionnaire (Cammann et al., 1979) with a 7-point Likert-type scale ranging from 1 (strongly disagree) to 7 (strongly agree). It consists of three items (e.g., “All in all, I am satisfied with my job.”). Good psychometric properties of the scale have been found in several studies (e.g., Cammann et al., 1979; Bowling and Hammond, 2008).

All reliability coefficients were satisfactory and are displayed in Table 1.

## Statistical Analyses

In order to evaluate the psychometric properties of the scores from the German version of the JCQ, we evaluated the data’s internal structure, estimated the scores’ reliability, and tested a selected relation within the nomological network. Moreover, measurement invariance between the German and English versions of the JCQ was analyzed. Finally, we compared the correlations between the job crafting score and a score for job satisfaction in our sub-samples. For testing the structural equation models, the full information maximum likelihood approach (FIML) was used to deal with missing values. This approach involves estimating a likelihood function using all available data from a case (Rosseel et al., 2018).

### Evidence for Validity Based on Testing of the Internal Structure

The internal structure was examined by comparing the fit of the theoretically assumed three-factor structure with the fits for a one-factor and a bifactor model of job crafting in both datasets using structural equation modeling with maximum likelihood (ML) estimation using R (Version 3.3.0, R Core Team, 2016) and the lavaan package (Rosseel et al., 2018). Before starting the analyses, we tested the multivariate normality requirements for the use of ML estimators by means of Mardia’s Test (MVN-package, Korkmaz et al., 2014). The results of Mardia’s Test supported the assumption of multivariate normality (multivariate skewness = 13.75, *p* = 0.185, multivariate kurtosis = 0.37, *p* = 0.715). As bifactor models often fail to converge or often yield incorrect parameter estimates, we followed the recommendations by Eid et al. (2017) and specified a bifactor-(S⋅I – 1). In such a model, one item is used to identify the bifactor and is excluded from specifying the facet it belongs to.

To evaluate model fit, we considered the exact model fit using the χ^2^ goodness-of-fit statistic. Additionally, we considered the Root Mean Square Error of Approximation (RMSEA), the Comparative Fit Index (CFI), and Standardized Root Mean Residual (SRMR), using the cutoff criteria proposed by Hu and Bentler (1999) as well as Beauducel and Wittmann (2005) (RMSEA ≤ 0.06, CFI ≥ 0.95, and SRMR ≤ 0.08). The use of goodness of fit indices and their cutoff –criteria (e.g., Hu and Bentler criteria) with questionnaire data has been criticized for not sufficiently detecting misspecifications (Greiff and Heene, 2017). The authors summarized that the results of model fit measures can be influenced by circumstantial variables that are not related to the model fit. Therefore, the recommended cutoff-criteria should be applied carefully and not as “golden rules” (Heene et al., 2011). Considering that, we followed suggestions proposed by Heene et al. (2011). In addition to applying goodness of fit indices, they recommended modeling and interpreting sources of misfit. In case that the specified model did not fit the data well, misfit was investigated and modeled to ensure that the parameter interpretations were not flawed.

The analyses comprised two steps. First, the three individual measurement models (task, cognitive, and relational crafting) were evaluated. If the measurement models showed a considerable misfit, the modification indices were inspected, and the measurement models were re-specified in accordance with Heene et al. (2011). Importantly, only theoretically feasible modifications were allowed. In the second step, the hierarchical three-factor structure of job crafting was tested. Moreover, a one-factor and a bifactor model of job crafting were also evaluated and compared with the theoretically assumed three-factor model, including a second-order general factor above the three facets, using Akaike (AIC) and Bayesian information criteria (BIC) as well as a chi-square difference test. Lastly, to ensure completeness, the three-factor structure was evaluated in the Australian sample.

### Reliability Estimates

To estimate reliability, several reliability coefficients were used. McDonald’s omega was estimated in order to provide a means of comparing the German and the original version of the JCQ. Moreover, Cronbach’s alpha (only for the facets of job crafting) and test–retest reliability (Pearson correlation) were estimated.

### Evidence for Validity Based on Relations With Other Variables

To test parts of the nomological network as source of validity evidence, we used product-moment correlations between the job crafting scores and scores of tests measuring similar, antecedent, and consequent constructs. As a means of evaluating the precision of these estimates, 95% confidence intervals were taken into account.

### Evaluation of Measurement Invariance

We followed the recommendations by Meredith (1993) and Chen et al. (2005) and used a stepwise approach to test configural, factor loading, and intercept invariance. To evaluate the differences in fit between the subsequent levels of measurement invariance, the cut-off values proposed by Chen (2007) were used. Accordingly, for values of ΔCFI > 0.010, ΔRMSEA > 0.015, Δ SRMR > 0.030 (Δ SRMR > 0.010 for comparing factor loading with intercept invariance), measurement invariance cannot be assumed. Additionally, a chi-square difference test was used. Before measurement invariance could be evaluated, the job crafting items in the German and the Australian dataset had to be z-standardized, because different rating scales were used in both test versions.

#### Relation With Job Satisfaction

We used structural equation modeling to include the relation between job crafting and job satisfaction within the nomological network in the German and Australian subsamples as an addition to the classical invariance testing procedure. In particular, we added the job satisfaction item to the preferred model and correlated it with the latent job crafting variable. As the Australian dataset did not include the job satisfaction item, we took the reported correlation (*r* = 0.43) and the estimated construct reliability for job crafting (0.79, see below) and estimated the disattenuated correlation in the Australian sample [0.43/sqrt(0.79)]. This value was then used to fix the correlation between job crafting and job satisfaction in the German sample. Finally, we compared a model without the fixed correlation and a model with the fixed correlation. If the translated version of the questionnaire worked, the model with the fixed correlation should not differ significantly. In other words, the relation found in Australia should also be found in Germany. Again, we used the criteria suggested by Chen (2007) to choose the best-fitting model.

## Results

### Missing Values

In both the German and the Australian datasets, missing values were found. The full information maximum likelihood approach (fiml) as implemented in lavaan (Rosseel, 2012) was used to treat missing values. Most of the missings in the German dataset and all of the missings in the Australian dataset were at least missing at random (Enders, 2010). However, for a subgroup within the German dataset, the missings were systematic: A total of 16 participants in the German dataset were self-employed without any staff members. Their potential to engage in relational and cognitive job crafting was partly limited in comparison to employees with co-workers and company affiliation. Therefore, these self-employed participants did not fill in four relational crafting items (Relational Crafting Items 1, 3, 4, 5; see Appendix C) and one cognitive crafting item (Cognitive Crafting Item 2; see Appendix C). These systematic missing values (not missing at random) violated the conditions of the fiml approach. Therefore, we conducted all analyses with and without the 16 self-employed participants and compared the results. We found that the results did not differ when omitting the cases in question. We conclude that the data of the self-employed participants did not distort the results and therefore we did not exclude them from our analyses.

### Evidence for Validity Based on Testing the Internal Structure

The results of the evaluation of all models are displayed in Table 2. The measurement models for task and relational crafting demonstrated acceptable fits. However, a considerable misfit was found for the measurement model for cognitive crafting. According to the modification indices, several items shared variance that went beyond cognitive crafting. Due to wording or content similarities, the residuals of the Cognitive Crafting Items 1 and 4, Items 2 and 3, and Items 1 and 5 were allowed to correlate. All items are listed in Appendix C. After re-specification by permitting correlated residuals, the cognitive crafting measurement model demonstrated an adequate fit. Furthermore, the results showed an adequate fit for the theoretically assumed hierarchical three-factor model. The one-factor model yielded a considerable misfit, and both AIC and BIC were larger than in the three-factor model. The bifactor model yielded a better model fit than both other models. However, the construct reliability for the relational crafting facet was only ω = 0.19. In addition, the same facet also had a negative loading which was most likely due to estimation problems (Eid et al., 2017). Consequently, the results supported the proposed hierarchical three-factor structure, as found by Slemp and Vella-Brodrick (2013).

**Table 2:** Measurement models, evaluation of validity of internal structure, and measurement invariance testing.

Models	c^2^/df (*p*)	Δc^2^/Δdf (*p*)	CFI	DCFI	RMSEA (CI 90%)	DRMSEA	SRMR	DSRMR	AIC	BIC
**Measurement models**										
Task crafting	11.316/5 (0.045)		0.986		0.051 (0.007–0.091)		0.022			
Cognitive crafting	77.008/5 (<0.001)		0.882		0.173 (0.140–0.208)		0.058			
Relational crafting	10.153/5 (0.071)		0.988		0.046 (0.000–0.087)		0.021			
Cognitive crafting (re-specified)	2.331/2 (0.312)		0.999		0.019 (0.000–0.094)		0.009			
**Evaluation of validity of internal structure**										
Australian three-factor model	216.509/87 (<0.001)		0.950		0.067 (0.056–0.078)		0.050		15067.908	15250.699
Australian bifactor model	162.703/73 (<0.001)	53.806/14 (<0.001)	0.966		0.061 (0.048–0.073)		0.037		15042.103	15278.208
Australian one-factor model	983.355/90 (<0.001)	766.846/3 (<0.001)	0.657		0.173 (0.163–0.182)		0.109		15828.755	16000.121
German three-factor model	236.781/84 (<0.001)		0.910		0.061 (0.052–0.071)		0.061		17567.583	17780.658
German bifactor model	149.848/70 (<0.001)	86.933/14 (<0.001)	0.953		0.049 (0.038–0.059)		0.041		17508.650	17180.217
German one-factor model	675.688/87 (<0.001)	438.907/3 (<0.001)	0.654		0.118 (0.110–0.127)		0.095		18000.490	18201.031
**Measurement invariance testing**										
Configural model	453.290/171 (<0.001)		0.934		0.064 (0.057–0.071)		0.057		30321.997	30787.613
Factor loading invariance model	488.011/185 (<0.001)	34.721/14 (0.002)	0.930	0.004	0.063 (0.057–0.070)	0.001	0.065	−0.008	30328.718	30728.489
Intercept invariance model	488.078/196 (<0.001)	0.067/11 (>0.999)	0.932	−0.002	0.060 (0.054–0.067)	0.003	0.065	<0.001	30306.785	30654.821

*c^2^, chi-square goodness-of-fit statistic; CFI, comparative fit index; RMSEA, root mean square error of approximation; CI, 90% confidence interval; SRMR, standardized root mean square residual; BIC, Bayesian Information criterion; AIC, Akaike information criterion. For tests of invariance, each model was compared against the less restrictive model listed in the row above based on differences in CFI, RMSEA, and SRMR. Additionally, the c^2^-difference test was used to compare the different factor models and to compare the invariance testing models.*

### Reliability Estimates

Cronbach’s alpha was adequate for the individual job crafting facets (see Table 1 for all reliability coefficients). Because of the hierarchical structure of job crafting, we estimated McDonald’s omega. McDonald’s omega was also adequate for the individual job crafting facets and the whole job crafting scale. Test–retest reliability was also adequate, but slightly lower than the internal consistency estimates. Additionally, item analyses were conducted with the psychometric package (Fletcher and Fletcher, 2013) in R and results can be found in Appendix D.

### Evidence for Validity Based on Relations With Other Variables

As expected, the job crafting score correlated positively with scores for similar, antecedent, and consequent constructs (i.e., personal initiative, autonomy, creative self-efficacy, vigor, and job satisfaction; see Table 1). Furthermore, we inspected product-moment correlations between job crafting scores and related constructs on the level of the three facets of job crafting. The positive correlations between the job crafting score and scores for associated constructs that had been found on an overall level also manifested themselves on the facet level for personal initiative, creative self-efficacy, vigor, and job satisfaction. However, the correlation pattern for the autonomy score was different, showing no significant correlation with the cognitive crafting score.

### Measurement Invariance

All results of measurement invariance testing are displayed in Table 2. The differences in RMSEA, CFI, and SRMR between all three tested invariance models were below the cut-off criteria for measurement invariance proposed by Chen (2007). Thus, the latent means could be compared across groups (Chen et al., 2005). The estimated latent-mean differences between the German and Australian datasets were 0.006 (*p* = 0.875, *d* = 0.012) for task crafting, 0.008 (*p* = 0.827, *d* = 0.014) for cognitive crafting, 0.007 (*p* = 0.863, *d* = 0.013) for relational crafting, and 0.011 (*p* = 0.743, *d* = 0.024) for overall job crafting. Thus, there was no significant difference between the latent means in the German and Australian datasets.

#### Relation With Job Satisfaction

Generally, as can be seen in Table 1, the job crafting scores correlated positively with the score for job satisfaction. However, as explained above, we included this key relation in our invariance testing. Since it fitted the data best, we used the hierarchical three-factor model of job crafting. The model without a fixed correlation between job crafting and job satisfaction yielded an acceptable fit: χ^2^(98) = 312.574, *p* < 0.001, CFI = 0.882, RMSEA = 0.067 (CI: 0.059–0.076), SRMR = 0.063. The model in which this key relation was fixed to the disattenuated Australian correlation fitted slightly better: χ^2^(99) = 281.439, *p* < 0.001, CFI = 0.894, RMSEA = 0.062 (CI: 0.053–0.070), SRMR = 0.071. The differences were above the cut-offs by Chen, suggesting that the key relation is indeed invariant. Furthermore, a chi-square difference test indicated a better model fit for the model with the fixed correlation: Δc^2^ = 31.134, Δdf = 1, *p* < 0.001.

Both models did not yield ideal fits. As recommended by an reviewer, we also specified correlations between the facet residuals and job satisfaction. However, this did not improve the model fit: χ^2^(95) = 311.11, *p* < 0.001, CFI = 0.881, RMSEA = 0.069 (CI: 0.060–0.077), SRMR = 0.063 for the model without fixed correlation. Looking at the modification indices shows that the model can only be improved by specifying the relations between item residuals and the criterion.

## Discussion

In this study we aimed to produce and evaluate a German translation of the Job Crafting Questionnaire (JCQ) by Slemp and Vella-Brodrick (2013), which is invariant to the original measure. We demonstrated first evidence that the proposed three-factor structure was also apparent in the German JCQ and found support for the reliability of the test score interpretations. Furthermore, we tested the theoretically meaningful relationships between job crafting and related constructs within the nomological network of job crafting. In sum, these results indicate acceptable psychometric properties of the German JCQ. Moreover, tests of measurement invariance using the German sample and the original Australian sample of Slemp and Vella-Brodrick (2013) indicate that the JCQ is an invariant measurement of job crafting across German- and English-speaking samples. Finally, we extended the classic measurement invariance procedure by showing that the correlations between overall job crafting and job satisfaction did not differ between the samples.

### Evidence for Validity Based on Testing the Internal Structure

The initial inspection of the measurement models revealed a considerable misfit in the cognitive crafting model. The cognitive crafting model was re-specified by permitting correlated residuals between items that shared wording and had similar content, leading to an adequate fit. Subsequently, an evaluation of the three-factor structure in the German dataset indicated that a model consisting of the three job crafting facets described our data adequately. Based on our testing of the hierarchical three-factor model against different one-factor or bifactor models and from inspection of the loadings of the items, we infer that the theoretically assumed hierarchical three-factor model fits the data best.

### Reliability Estimates

Cronbach’s alpha was satisfactory, but it was descriptively lower than the Cronbach’s alpha of the original JCQ facets (Slemp and Vella-Brodrick, 2013). However, as reliability is a sample-dependent measure, it does not necessarily reflect the quality of the test used. Moreover, McDonald’s omega was satisfactory for the facets as well as for the overall job crafting factor. Test–retest reliability for overall job crafting was adequate. For the individual job crafting factors, the test–retest reliability was descriptively lower than the internal consistency measures and slightly lower than 0.70. This result might be due to stability aspects of job crafting. In support of this assumption, Wrzesniewski and Dutton (2001, p. 180) defined job crafting as a “situated activity, in the sense that different contexts enable or disable different levels and forms of crafting.” Thus, contextual aspects might limit the possibility of engaging in job crafting at times, diminishing the stability of job crafting. In conclusion, the German JCQ demonstrates adequate reliability.

### Evidence for Validity Based on Relations With Other Variables

In line with our hypotheses, job crafting correlated positively with personal initiative, autonomy, creative self-efficacy, vigor, and job satisfaction. Based on an inspection of the correlations between the facets of job crafting and associated constructs, we can conclude that the relationships manifest themselves equally on the facet level. However, there was one exception: Autonomy did not show a significant relationship with cognitive crafting. This result seems explainable as autonomy was operationalized as work-scheduling autonomy in the scope of this paper. Hence, freedom about the timing of tasks might not strongly influence whether employees perceive their work meaningful.

Furthermore, we introduced a new construct within the nomological network of job crafting, namely creative self-efficacy. Our results showed that job crafting is positively associated with creative self-efficacy. This result is in line with the assumptions of Wrzesniewski and Dutton (2001) who describe job crafting as the creative process of shaping one’s work. Thus, job crafting is not only correlated with general self-efficacy (Niessen et al., 2016), but also with domain-specific creative self-efficacy. This result broadens the nomological network of job crafting by including the concept of creativity.

All in all, the results give first evidence of validity based on relations with other variables, which were theoretically derived from the nomological network.

### Measurement Invariance

Despite some differences in sample composition regarding age, employment type, and working hours per week, intercept invariance (strong factorial measurement invariance) could be established. Thus, it can be assumed that these variables do not affect the way job crafting manifests. Furthermore, “the unit of measurement of the underlying factor is identical” (Chen et al., 2005, p. 474) in the different test versions and consequently they measure the same construct. Moreover, both test versions offer the same scale origins and therefore the latent means can be compared across groups (Widaman and Reise, 1997; Chen et al., 2005). The comparison of latent means between the German and Australian samples in our datasets revealed no significant differences for job crafting and its facets. Consequently, in the samples at hand, Germans and Australians demonstrated the same levels of job crafting. To the best of our knowledge, only two studies have tested job crafting measures to be invariant across cultures (Gordon et al., 2015; Nielsen et al., 2017). However, Nielsen et al. (2017) showed a lack of factor loading invariance when they compared a Chinese sample with a British or Spanish sample. Factor loading invariance is needed to compare correlations within the nomological network. Furthermore, the authors find a lack of intercept invariance across any samples. Gordon et al. (2015) did not test for intercept invariance across samples. However, intercept invariance is needed to compare latent means (Sass, 2011). In conclusion, the JCQ allows for comparisons between German- and English-speaking samples and represents a useful tool for intercultural research questions concerning job crafting.

Our proposal to select key relations from the nomological network that should not differ between the samples used for invariance testing is also of importance. We applied this approach to the relation between job crafting and job satisfaction in this study. As explained above, the cultural values in Australia and Germany that are relevant for this relation do not differ. Thus, the relation should be invariant. This was indeed what we found. By adding this step to the classic procedure of invariance testing, we believe that the test generates results, which are even more reliable than the classic approach. Moreover, in some cases, researchers must deal with partial invariance. Here, it is always unclear how strongly the analyses based on partially invariant models are distorted by the lack of invariance of some model parameters. By including the proposed additional step of adding a key relation to the invariance testing, we address this insecurity. Clearly, much more research using simulated and real data is necessary. However, this paper shows the ease of adding this step and also sets out the advantages gained from doing so.

### Theoretical and Practical Implications

In our stepwise statistical procedure, we tested for evidence supporting the validity of internal structure and reliability of the scores, established and tested parts of a nomological network as source of evidence for validity based on relations to other variables, designed a validation plan, and tested the hypotheses accordingly. Afterwards we tested for measurement invariance, which allowed us to compare job crafting and relations within its nomological network across Germany and Australia. We learned several lessons from this procedure. In sum, cultural differences do not influence the good psychometric properties and the measurement invariance of the JCQ. Even though the JCQ is invariant across cultures and the extent of job crafting behavior is the same in Germans and Australians, it is important to compare key correlations such as job satisfaction as they give further information about the nature and effects of job crafting in different cultures as well as about potential problems in the test construction.

Furthermore, we found a positive relationship between antecedent creative self-efficacy and job crafting, broadening the nomological network of job crafting. Although creativity plays a role in the job crafting model (Wrzesniewski and Dutton, 2001), no former study has investigated this relationship yet. Our study supports the importance of creativity within the nomological network of job crafting.

There is also a practical implication of our study. Work settings are becoming more international. In Germany the percentage of foreign employees has risen to nearly 10% (Crößmann and Mischke, 2016). Hence, cultural differences in proactive work behavior, such as job crafting have sparked an interest among several researchers and practitioners. Oldham and Fried (2016) identified a need for invariant measures of this behavior in different languages and cultures. Our study makes a first contribution toward meeting this empirical need and provides a measurement that is invariant across German- and English-speaking samples that allows a comparison of job crafting within multicultural work settings.

### Limitations

There are several limitations to this study. The rating scales used in the German and the original version of the JCQ differed. In the German version, a 5-point Likert scale from 1 (never) to 5 (very often) was used, whereas a 6-point Likert scale from 1 (hardly ever) to 6 (very often) was used in the original Australian version. Although we z-standardized job crafting data before performing measurement invariance evaluations, the different rating formats are problematic, as they might be interpreted differently by participants. The different number of scale points might convey different impressions of the resolution of the scale. Moreover, a 5-point scale has a neutral category in comparison to a 6-point scale. This might lead to a tendency to choose the neutral alternative in the German sample. Moreover, different labeling of the lower scale point might also impact interpretations of the scale and the answering behavior of the participants.

A second limitation of the study is that both the German and the Australian samples were convenience samples. These samples may be subject to range restrictions, clumping, or neglecting of subgroups. In the future, it would be good to have more representative samples that allow stronger generalizations of the study results.

Another limitation of this study is that only a small part of the nomological network of job crafting could be tested. Future research could further investigate relationships between job crafting assessed with the German JCQ and other associated constructs. Especially research on the links to constructs proposed in the job crafting model by Wrzesniewski and Dutton (2001) could be of interest.

Additionally, we only used cross-sectional data to test relations within the nomological network of job crafting. Therefore, no causal inferences can be drawn. Future research should address this issue by using longitudinal data to test antecedences and consequences of job crafting.

Furthermore, we must note that the assessment of job satisfaction differed in both samples. Thus, comparisons of relations could be more difficult. For a better informative value, we recommend researchers to compare relations derived from the nomological net using constructs assessed with the same measuring instrument.

Finally, the reliability estimates of the German version JCQ scores were adequate but descriptively lower than those of the Australian original. Future research could address this issue by evaluating the reliability of the German JQC in different samples and by further investigating the stability of job crafting.

## Conclusion

Based on the results of our stepwise procedure of testing psychometric properties and invariance, we can conclude that the scores derived from the German JCQ give first evidence of internal structure validity and reliability. Relationships within the nomological network of job crafting were confirmed. As intercept invariance between the German version and the Australian original was found, comparisons between German- and English-speaking samples are possible. The comparisons of relations between job crafting and job satisfaction demonstrated the advantages of bolstering the classic invariance testing approach by adding relations that are also assumed to be invariant. Finally, the German JCQ is a useful tool for research in the field of work psychology as well as for businesses that want to utilize the benefits of job crafting for (multicultural) organizations.

## Ethics Statement

The study was conducted in accordance with the Declaration of Helsinki (World Medical Association, 2013). The ethics committee of Institute of Psychology of Humboldt University of Berlin, Germany approved the German study design and the German questionnaires regarding data protection and compliance with ethical guidelines. The Monash University Human Research Ethics Committee (MUHREC), Australia, approved the Australian study design and the Australian questionnaires regarding data protection and compliance with ethical guidelines.

## Author Contributions

VS and EC designed the research and collected the data. VS, SE, and MZ carried out the data analysis and interpretation. VS and SE carried out the research on the theory and drafted the manuscript. MZ, EC, AH, and GS conducted critical revision. GS collected and provided the data of Slemp and Vella-Brodrick (2013). All authors read and approved the final manuscript.

## Conflict of Interest Statement

The authors declare that the research was conducted in the absence of any commercial or financial relationships that could be construed as a potential conflict of interest.
