# Supplementary material for: Measuring Job Crafting Across Cultures: Lessons Learned From Comparing a German and an Australian Sample
Source: Front Psychol. 2019 May 7;10:991. doi: 10.3389/fpsyg.2019.00991 (PMC6514196; doi:10.3389/fpsyg.2019.00991)
Supplement: Supplementary file 1 [file Data_Sheet_1.pdf]

1 Appendix A

Table A1. Differences between measurement waves 1 and 2

	t	*df*	*p*	*g*
Age	2.98	119	.003	.31
Job Crafting	.40	120	.693	.04
Task Crafting	3.42	120	<.001	.35
Cognitive Crafting	.40	120	.688	.04
Relational Crafting	2.47	120	.015	.25
Personal Initiative	2.29	121	.024	.23
Autonomy	.81	122	.422	.08
Creative Self-Efficacy	.27	121	.785	.03
Vigor	1.61	122	.110	.16
Job Satisfaction	.89	122	.373	.09

Note. The t-test for dependent samples was performed. Hedge’s g was used as a measure of effect size.

2 Appendix B

Table B1. Comparison between demographic variables of the German and Australian samples

Construct	German sample	Australian sample	Difference between the samples	Effect size
Age	*M* = 37.87 (*SD* = 10.68) range: 19 - 72 years	*M* = 41.95 (*SD* = 11.36) range: 23 - 71 years	$t(481) = 4.61, p < .001$	$g = .37$
Gender	59 % female 36% male 5 % no comment	68 % female 32 % male	$\chi^2(1) = 2.16, p = .14$	$V = .06$
Employment type	83% full-time 17% part-time	74% full-time 26% part-time	$\chi^2(1) = 7.63, p < .01$	$V = .11$
Working hours per week	*M* = 42.06 (*SD* = 12.21)	*M* = 37.95 (*SD* = 12.13)	$t(506) = 4.32, p < .001$	$g = .34$

Note. Hedge's g was used as a measure of effect size for t-tests. Cramer's V was used as a measure of effect size for chi-square tests of independence.

3 Appendix C

Table C1. Job crafting items in the English and German languages

Item	In English Language (Slemp & Vella-Brodrick, 2013)	German Translation
Task Crafting		
1	Introduce new approaches to improve your work.	Ich führe neue Vorgehensweisen ein, um meine Arbeit zu verbessern.
2	Change the scope or types of tasks that you complete at work.	Ich verändere den Aufgabenbereich oder die Art der Aufgaben, die ich während der Arbeit erledige.
3	Introduce new work tasks that you think better suit your skills or interests.	Ich führe neue Aufgaben ein, die besser zu meinen Fertigkeiten und Interessen passen.
4	Choose to take on additional tasks at work.	Ich entscheide mich, bei der Arbeit zusätzliche Aufgaben zu übernehmen.
5	Give preference to work tasks that suit your skills or interests.	Ich bevorzuge Arbeitsaufgaben, die zu meinen Fertigkeiten und Interessen passen.
Cognitive Crafting		
1	Think about how your job gives your life purpose.	Ich denke darüber nach, wie meine Arbeit meinem Leben Sinn gibt.
2	Remind yourself about the significance your work has for the success of the organization.	Ich rufe mir in Erinnerung, wie wichtig meine Arbeit für den Erfolg der Organisation ist.
3	Remind yourself of the importance of your work for the broader community.	Ich rufe mir in Erinnerung, wie bedeutsam meine Arbeit für die Gesellschaft ist.
4	Think about the ways in which your work positively impacts your life.	Ich denke darüber nach, wie meine Arbeit mein Leben positiv beeinflusst.
5	Reflect on the role your job has for your overall well-being.	Ich reflektiere darüber, welche Rolle meine Arbeit für mein allgemeines Wohlbefinden spielt.
Relational Crafting		
1	Make an effort to get to know people well at work.	Ich bemühe mich, die Leute bei der Arbeit gut kennenzulernen.
2	Organise or attend work related social functions.	Ich organisiere oder besuche arbeitsbezogene gesellschaftliche Veranstaltungen.
3	Organise special events in the workplace (e.g., celebrating a co-worker's birthday).	Ich organisiere Sonderveranstaltungen bei der Arbeit (z.B. die Geburtstagsfeier eine/r Kollege/in).
4	Choose to mentor new employees (officially or unofficially).	Ich entscheide mich, neue Mitarbeiter/innen zu betreuen (offiziell oder inoffiziell).
5	Make friends with people at work who have similar skills or interests.	Ich freunde mich mit Leuten bei der Arbeit an, die ähnliche Fertigkeiten oder Interessen besitzen.

Note. Items in the original Job Crafting Questionnaire were rated on a 6-point Likert-type scale from *hardly ever* to *very often*. Items of the German version of the Job Crafting Questionnaire were rated on a 5-point Likert-type scale from *never* to *very often*.

17 Appendix D

Table D1. Item statistics of job crafting items in the German dataset

Item i	Task Crafting			Cognitive Crafting			Relational Crafting			Job Crafting
	$\bar{x}_i$	SD_i	$r_{i(t-i)}$	$\bar{x}_i$	SD_i	$r_{i(t-i)}$	$\bar{x}_i$	SD_i	$r_{i(t-i)}$	$r_{i(t-i)}$
TC 1	3.30	.76	.49							.38
TC 2	2.99	.89	.57							.31
TC 3	2.75	.94	.62							.45
TC 4	3.37	.78	.41							.44
TC 5	4.05	.75	.19							.21
CC 1				3.52	.92	.57				.39
CC 2				2.90	.99	.42				.41
CC 3				2.86	1.11	.55				.41
CC 4				3.38	.93	.62				.52
CC 5				3.67	.81	.49				.42
RC 1							3.65	.93	.54	.46
RC 2							3.04	.96	.52	.44
RC 3							2.35	1.04	.53	.44
RC 4							3.09	1.12	.42	.38
RC 5							3.49	.94	.44	.43

Note. For rating scales the mean ($\bar{x}_i$) is used to express the psychometric difficulty of items. The rating scale used ranges from 1 to 5. SD_i = standard deviation of items; $r_{i(t-i)}$ = correlation of item with total test score (scored without item).
